# Subtropical southern Africa fire emissions of nitrogen oxides and ammonia obtained with satellite observations and GEOS-Chem†

**DOI:** 10.1039/d5ea00041f

**Published:** 2025-06-16

**Authors:** Eloise A. Marais, Martin Van Damme, Lieven Clarisse, Christine Wiedinmyer, Killian Murphy, Guido R. van der Werf

**Affiliations:** a Department of Geography, University College London London UK e.marais@ucl.ac.uk; b Spectroscopy, Quantum Chemistry and Atmospheric Remote Sensing (SQUARES), BLU-ULB Research Center, Université libre de Bruxelles (ULB) Brussels Belgium; c Royal Belgian Institute for Space Aeronomy (BIRA-IASB) Brussels Belgium; d Cooperative Institute for Research in Environmental Sciences, University of Colorado Boulder Boulder CO USA; e Wolfson Atmospheric Chemistry Laboratories, Department of Chemistry, University of York York UK; f Meteorology & Air Quality Group, Wageningen University and Research Wageningen The Netherlands

## Abstract

Landscape fires in subtropical southern Africa (2–20°S) are a prominent regional source of nitrogen oxides (NO_*x*_) and ammonia (NH_3_), affecting climate and air quality as precursors of tropospheric ozone and aerosols. Here we evaluate GEOS-Chem model skill at reproducing satellite observations of vertical column densities of NO_2_ from TROPOMI and NH_3_ from IASI driven with three distinct and widely used biomass burning inventories (FINNv2.5, GFEDv4s, GFASv1.2). We identify that GFASv1.2 use of fire radiative power and a NO_*x*_ emission factor that is almost half that used by the other two inventories is most consistent with TROPOMI and that FINNv2.5 use of active fires and landscape-specific fuel loads and biomass consumed is most consistent with IASI. We use a simple mass-balance inversion to calculate top-down NO_*x*_ emissions of 1.9 ± 0.6 Tg NO for June–October and NH_3_ emissions of 1.2 ± 0.4 Tg for July–October. All inventories collocate NO_*x*_ and NH_3_ emissions, whereas most of the pronounced emissions of NO_*x*_ and NH_3_ are separate and have distinct seasonality in the top-down estimate. We infer with GEOS-Chem more efficient ozone production (13 Tg ozone per Tg NO) with the top-down informed NO_*x*_ emissions than the inventory emissions, as GFASv1.2 NO_*x*_ is almost 20% less than top-down NO_*x*_ and the 2.3- to 2.5-times greater FINNv2.5 and GFEDv4s NO_*x*_ reduces sensitivity of ozone formation to NO_*x*_. Both NO_*x*_ and NH_3_ top-down emissions are unaffected by use of plume injection heights, limited to GFASv1.2 in GEOS-Chem, and NH_3_ is insensitive to acidic sulfate and nitrate aerosol emissions absent in all inventories. The top-down emissions estimates and comparison to satellite observations suggest a hybrid bottom-up approach could be adopted to discern byproducts of smouldering and flaming fires.

## Abstract 2

Observationally-informed top-down estimates of emissions of nitrogen oxides and ammonia for the dry burning season of one of the most fire-prone regions in the world used to assess widely used biomass burning inventories.

## Graphical abstract

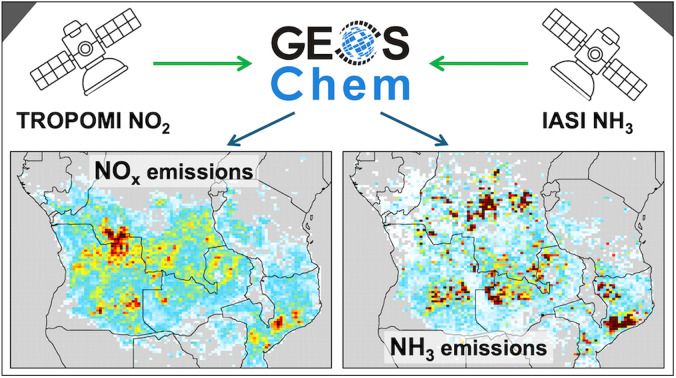

**Environmental significance**

Subtropical southern Africa is the most fire-prone region in the world, emitting large amounts of reactive nitrogen as nitrogen oxides (NO_*x*_) and ammonia (NH_3_). Uncertainties in bottom-up inventories impede assessment of the influence of this reactive nitrogen on air quality, climate, and atmospheric oxidants. We calculate observationally-informed emissions using satellite observations and a chemical transport model. We identify that no single inventory reproduces top-down emissions of both NO_*x*_ and NH_3_. All collocate the two, even though NO_*x*_ is from efficient combustion and NH_3_ from inefficient fires. We suggest plausible steps to resolve these issues for ease of use of existing inventories in models. We also advocate for ground-based monitoring to validate the datasets used to calculate top-down emissions.

## 1. Introduction

Open burning of biomass emits large quantities of the reactive nitrogen trace gases nitrogen oxides (NO_*x*_ ≡ NO + NO_2_) and ammonia (NH_3_).^1,2^ Both are directly hazardous to nitrogen-sensitive habitats and are precursors of aerosols that alter regional climate and affect public health.^3–5^ NO_*x*_ from biomass burning also leads to prompt and sustained formation of the greenhouse gas and air pollutant ozone.^6,7^ NO_*x*_ and NH_3_ are observable from space-based spectrometers as NO_2_ in the UV and NH_3_ in the infrared. Even though rapid urbanization and population growth is increasing urban sources of air pollution in subtropical southern Africa,^8^ vast open burning of biomass is still an overwhelmingly dominant local, dry season source of trace gases and aerosols. The burning season is longer (6 months) and biomass burned typically exceeds other prominent fire-prone regions, necessitating observationally-informed knowledge of the emissions and influences of NO_*x*_ and NH_3_ on local air quality, local and remote tropospheric ozone, and reflective aerosols.

The burning season in subtropical southern Africa (2–20°S) covers the very dry season from May to October. According to bottom-up inventories and satellite observations of fire datasets, burning migrates south during the dry season from near the Equator to southern Angola and the Mozambique coast.^9,10^ Ignition is by humans for agricultural practices such as crop residue burning, conversion of savannas to farmland, and production of biochar to fertilize soils.^11^ Fire propagation results from connectivity of the vast savanna landscape of dry grasses that burn easily.^12^ Land fragmentation by roads, urban settlements, and croplands slows the spread of fires,^12^ but this effect has so far mainly caused a discernible decline in regional burned area over the satellite record in northern Africa.^13^

The amount of biomass burned (∼670 Tg carbon (C) per year) in subtropical southern Africa is ∼30% of global landscape-burned biomass in 1997–2016 and exhibits relatively small interannual variability.^9^ For context, only the anomalously large fires in Equatorial Asia in 1997 surpassed subtropical southern Africa at ∼1100 Tg C over the same time period.^9^ Of more recent anomalous fires, the biomass burned is similar to carbon emissions from the 2023 fires in Canada at ∼650 Tg C^14^ and far greater than the 2019–2020 fires in Australia at ∼200 Tg C.^15^

Reactive nitrogen emissions from fires result from reduced nitrogen stored in plants, mostly as amides and amines.^16^ The proportion of NO_*x*_*versus* NH_3_ emitted varies with combustion efficiency. Greater combustion efficiency promoting oxidation of fuel nitrogen to NO_*x*_ results from high-temperature flaming fires, windy conditions, and dry fuel. Decline in combustion efficiency favouring formation of NH_3_ occurs for slow-burning smouldering fires, stable atmospheric conditions, and moist fuel.^16–18^ African savanna fires are dominated by the flaming regime,^19^ as the majority of vegetation burned in savanna landscapes is very flammable grass,^20^ though satellite observations support occurrence of smouldering fires too.^21,22^ Seasonality in satellite observations of NO_2_ and NH_3_ abundances suggest a transition from flaming to smouldering fires toward the end of the burning season, due to an increase in fuel moisture content. NO_2_ and burned area together peak a month earlier than NH_3_ concentrations and other indicators of inefficient combustion such as carbon monoxide (CO).^21^

Models used to determine the influence of biomass burning on atmospheric composition are driven with bottom-up inventories that calculate trace gas emissions as the product of the amount of dry matter burned (activity factor) and the rate of production of trace gases per mass of dry matter burned (emission factor). Activity factors are determined with satellite-derived data that provide information about fire timing, location, extent, intensity and persistence. These data include products such as fire counts, burned area, and fire radiative power. The first detailed compilation of emission factors published in 2001^23^ is routinely updated to incorporate additional measurements from laboratory and field experiments.^24–26^ Most measurements are of flaming fires^4,21^ and the emission factors in the inventories vary spatially with broad landcover types, but are temporally static. An examination of satellite observations of NO_2_ and indicators of combustion efficiency support greater temporal variability in emission factors caused by environmental conditions such as fuel nitrogen and moisture content.^22,27^

Ground-based observations for constraining biomass burning emissions of NO_*x*_ and NH_3_ are limited to historic intensive field campaign measurements of subtropical southern Africa fires from the 1990s and early 2000s,^28,29^ routine ozone and CO measurements on commercial aircraft that mostly sample long-range transported plumes with a distinct composition to plumes nearer fires,^7,30^ and networks of recently established low-cost air quality sensors that are concentrated in urban areas in Africa.^31,32^ Satellite observations offer daily global coverage of NO_2_ from the TROPospheric Monitoring Instrument (TROPOMI) and NH_3_ from the Infrared Atmospheric Sounding Interferometer (IASI). Retrieval products from both instruments are mature, have been widely used and error characterised, and include information to account for the vertical sensitivity of the instrument and prior assumptions about the vertical distribution of the retrieved trace gas for consistent comparison to models.^33–35^

Here we drive the GEOS-Chem chemical transport model with three distinct biomass burning inventories to evaluate the model against satellite observations of NO_2_ and NH_3_ for informed selection of the most suitable inventory for top-down estimates of subtropical southern Africa biomass burning NO_*x*_ and NH_3_ emissions. We go on to relate the top-down emissions to flaming *versus* smouldering fire regimes, to quantify the contribution of fires to ozone production and potential for long-range transport of NO_*x*_ in the form of peroxyacetyl nitrate (PAN), and to recommend how to address inconsistencies between inventories and our top-down estimates.

## 2. Methods

### 2.1 The biomass burning inventories

The biomass burning inventories we use are the Global Fire Emissions Database version 4 with small fires (GFEDv4s),^9^ the Fire Inventory from the National Center for Atmospheric Research (NCAR) version 2.5 (FINNv2.5),^36^ and the Copernicus Atmosphere Monitoring Service Global Fire Assimilation System version 1.2 (GFASv1.2).^37,38^ All three inventories follow the standard approach^39^ of calculating emissions (*E*) as the product of dry matter burned (DMB) and an emission factor (EF) (eqn (1)). GFEDv4s and FINNv2.5 calculate DMB as the product of area burned (*A*), above-ground biomass (*B*), and combustion completeness or proportion of biomass actually consumed (*α*) (eqn (2)),^39^ whereas GFASv1.2 calculates DMB as the product of fire radiative power and conversion factors that relate fire radiative power to DMB.^37^

1 *E* = DMB × EF

2 DMB = *A* × *B* × *α*

Each inventory uses distinct approaches and datasets to calculate inputs for eqn (1) and (2). GFEDv4s uses burned area (*A*) from the Moderate Resolution Imaging Spectroradiometer (MODIS). Small fires are absent in the burned area product, so are calculated with a parameterisation that uses small active fires detected by MODIS.^9^ Carbon burned is then calculated using a biogeochemical model that estimates fuels in each 0.25° gridbox based on carbon gains from photosynthesis and losses from respiration, herbivory and fires. Land cover and tree cover density information derived from satellites are used as input, and combustion completeness (*α*) are based on fuel classes and moisture conditions. Carbon burned is then converted to DMB using landcover specific total dry matter carbon mass calculated as the sum of Akagi *et al.*^25^ EFs of all carbon-containing trace gases and aerosols.

FINNv2.5 determines burned area (*A*) through geospatial processing of fire counts and ecosystem type.^36^ Fire counts are either obtained just with MODIS or with MODIS and Visible Infrared Imaging Radiometer Suite (VIIRS). We use the combined MODIS and VIIRS product that has enhanced detection of small fires due to the finer resolution of VIIRS (375 m) than MODIS (1 km).^36^ Vegetation density from the MODIS Vegetation Continuous Fields product, and ecosystem type from the MODIS Land Cover Type product are then used to derive *B*^36^ and *α*.^40^ GFASv1.2 uses MODIS fire radiative power and derives dry matter combustion rates using an earlier version (3.1) of GFED.^37^

Savanna vegetation dominates area burned in all three inventories. GFEDv4s and GFASv1.2 use a single landcover classification for savannas, whereas FINNv2.5 distinguishes this landcover type as savannas and as woody savannas.

The emission factors these inventories use for landcover relevant to the region, summarised in Table 1, are from Akagi *et al.*^25^ for GFEDv4s and a mix of Akagi *et al.*^25^ and a 2015 update (https://www.acom.ucar.edu/Data/fire/; last accessed 24 February 2025) for FINNv2.5. GFASv1.2 uses Andreae and Merlet^23^ emission factors for NH_3_ and an unpublished value for NO_*x*_. There are no reported emission factors for woody savannas, so FINNv2.5 uses values for chaparral vegetation that are included in the 2015 update to Akagi *et al.*^25^ FINNv2.5 emits NO_*x*_ as NO and NO_2_, whereas the others emit all NO_*x*_ as NO.

**Table 1:** Comparison of biomass burning inventory NO_*x*_ and NH_3_ emission factors

Vegetation type	Emission factor^a^ [g kg^−1^]		
	GFEDv4s	FINNv2.5^b^	GFASv1.2
**NO**_***x***_ **as NO**			
Tropical forest	2.55	2.6	2.3
Savanna	3.9	3.9	2.1
Woody savanna^c^	—	3.65	—

**NH**_**3**_			
Tropical forest	1.33	1.3	0.93
Savanna	0.52	0.56	0.74
Woody savanna^c^	—	1.2	—

a EFs in grams per kilogram DMB given in the same number of significant figures as reported in the inventory description papers for FINNv2.5 and GFASv1.2 and as used in GEOS-Chem for GFEDv4s.

b FINNv2.5 NO_*x*_ emitted as ∼50 (mol) % NO for savannas and ∼30% NO for woody savannas and tropical forests.

c Chapparal vegetation type EFs used by FINNv2.5 for woody savannas.

GFASv1.2 and FINNv2.5 are provided as daily emissions at 0.1° and GFEDv4s as monthly emissions at 0.25° with daily and 3-hourly scalings (also at 0.25°) to achieve finer temporal resolution. The 3-hourly scalings are produced by calculating climatological mean diel cycles of vegetation-specific fires from active fires detected by geostationary instruments over the Americas that are then extrapolated to other regions.^41^ GFASv1.2 distributes emissions vertically using daily mean altitude of maximum plume injection (or injection height) determined with the Plume Rise Model.^42^

### 2.2 The satellite observations

The UV-visible TROPOMI was launched into sun-synchronous orbit in October 2017. In 2019, our target year, the TROPOMI nadir pixel resolution increased from 7 km × 3.5 km to 5.5 km × 3.5 km on 5 August. The instrument achieves daily global coverage with a swath width of 2600 km and an equator crossing time of 13h30 local solar time (LST). We use Level 2 TROPOMI NO_2_ tropospheric columns from the Sentinel-5P Products Algorithm Laboratory (S5P-PAL) portal (https://data-portal.s5p-pal.com/; last acquired 30 January 2022). These are retrieved with algorithm version 02.03.01 that corrects for a low bias in NO_2_ over polluted scenes in previous versions.^43^ The latest available TROPOMI NO_2_ data version is 02.08.00, but the product updates mostly impact scenes covered with snow/ice.^44^ We filter the TROPOMI NO_2_ data to remove poor quality, cloud-contaminated pixels that have a quality flag < 0.75.^45^

We combine data from the infrared IASI instruments onboard MetOp-A and MetOp-B satellites launched to sun-synchronous orbit in October 2006 for MetOp-A and September 2012 for MetOp-B. Both instruments have daytime equator crossing times of 09h30 LST. IASI elliptical pixels are ∼12 km in diameter at nadir. As with TROPOMI, the wide swath (2200 km) enables daily global coverage. The IASI NH_3_ data product we use is Level 2 version 4.0.0 reanalysed Artificial Neural Network for IASI (ANNI)^33^ hosted on the AERIS data service (https://doi.org/10.25326/13; last accessed 6 January 2025). This is the first IASI ANNI version to include data needed to calculate averaging kernels.^33^ This enables recalculation of IASI NH_3_ total columns with local modelled *a priori* vertical profiles to mitigate influence of vertical sensitivity and prior assumptions of the vertical distribution of NH_3_ for comparison to models. Other product updates cause an average ∼20% increase in NH_3_ columns relative to the previous version for scenes with large NH_3_ enhancements.^33^ We use morning overpass data filtered to remove poor quality level 1 data and cloud-contaminated pixels (cloud fraction > 25%) identified with a provided prefilter quality flag value of zero.

### 2.3 The GEOS-Chem model

We simulate atmospheric concentrations of NO_2_ and NH_3_ for comparison to TROPOMI NO_2_ and IASI NH_3_ using GEOS-Chem model version 13.0.2 (https://zenodo.org/records/4681742, last acquired 12 April 2021) in its classical (GCClassic) configuration. We use the FlexGrid capability of the model to simulate a nested domain covering equatorial and subtropical southern Africa (21.5°S–2°N, 5–42°E) at 0.25° latitude × 0.3125° longitude (∼28 km × ∼34 km at the centre of the domain). At the boundaries, instantaneous trace gas and aerosol concentrations are updated every 3 hours from the same GEOS-Chem model version simulated at 4° × 5°. The model already includes GFEDv4s and GFASv1.2. FINNv2.5 emissions of trace gases and aerosols are added in this work using gridded daily emissions from the NCAR Research Data Archive (https://doi.org/10.5065/XNPA-AF09; last acquired 9 January 2025). We apply GFEDv4s daily and 3-hourly scaling factors to GFEDv4s monthly emissions. The same hourly scaling factors from the WRAP project^46^ are applied to FINNv2.5 and GFASv1.2, yielding peak emissions for both inventories at 1–5 pm, accounting for 68% of emissions, and minimum emissions at night, accounting for just 7% at 8 pm to 9 am local time. GFASv1.2 emissions are distributed vertically in the model by weighting the emissions in each gridbox by the height of each model layer up to the GFASv1.2 injection heights. All GFEDv4s and FINNv2.5 emissions are released to the lowest model layer.

Other NO_*x*_ and NH_3_ emissions in the model include NH_3_ emissions from soils, birds, and the ocean provided by the Global Emissions InitiAtive (GEIA) inventory^47^ and halved to address a well-known bias,^48–50^ lightning NO_*x*_ as described in Murray *et al.*,^51^ soil and fertilizer NO_*x*_ as described in Hudman *et al.*,^52^ and anthropogenic NO_*x*_ and NH_3_ from the global Community Emissions Data System (CEDS) inventory.^53^ The CEDS version we use includes scaling of emissions in Africa by McDuffie *et al.*^54^ to match the Diffuse and Inefficient Combustion Emissions in Africa (DICE-Africa) regional inventory.^55^ All the non-biomass-burning emissions are the same in the three model simulations, except for small differences in soil NO_*x*_ emissions due to dependence of these on nitrogen deposition resulting from differences in biomass burning emissions.

The model uses offline NASA GEOS-FP meteorology and includes detailed gas-phase and heterogeneous chemistry. Partitioning of NH_3_ to aerosols to form ammonium nitrate (NH_4_NO_3_) is determined with the ISORROPIA-II thermodynamic equilibrium model.^56^ The model chemistry is initialized with spin-ups of 1 month for the nested model and a year for the global boundary conditions. The model is sampled during June–October 2019 to encompass months when burned area peaks (June–September)^9^ and when emissions of CO and NH_3_ from declining combustion efficiency peak (August–October).^21,22^ The three outer boxes of the nested domain, the buffer zone, are influenced by the coarse resolution boundary conditions, so are ignored.

### 2.4 Comparison of the satellite observations and GEOS-Chem

Model and satellite observation coincidence is achieved by sampling the model at 13–14 LST to compare to TROPOMI and 9–10 LST to compare to IASI. To mitigate dependence of the comparison between modelled and TROPOMI tropospheric NO_2_ columns on vertical sensitivity of TROPOMI, we apply the TROPOMI averaging kernels to GEOS-Chem vertical profiles of NO_2_ using eqn (S1) and (S2) in the ESI.† ^57^ This is done by identifying coincidence between TROPOMI pixels and GEOS-Chem, interpolating the TROPOMI tropospheric averaging kernels to the GEOS-Chem vertical grid, and applying these regridded averaging kernels to the model.

The IASI retrieval relies on a single, fixed prior vertical profile for land and for ocean scenes, so consistent comparison between IASI and GEOS-Chem is achieved by reprocessing daytime IASI NH_3_ columns with local profiles from GEOS-Chem. Detailed descriptions of the reprocessing procedure are in Clarisse *et al.*^33^ for general application to IASI version 4 products and in Zhai *et al.*^58^ for first use of the averaging kernels with GEOS-Chem for IASI observations of PAN. These steps are summarised in the ESI (eqn (S3) and (S4)).† We screen the reprocessed IASI NH_3_ columns for retrievals with limited or no sensitivity to NH_3_ and that are either very noisy or are incompatible with spectral enhancements attributable to NH_3_.^33,58^

TROPOMI, IASI, and GEOS-Chem are all compared on the GEOS-Chem horizontal grid.

### 2.5 Mass-balance inference of emissions

We infer 24 h monthly NO_*x*_ and NH_3_ emissions in each 0.25° × 0.3125° box using a mass balance approach:^50^3
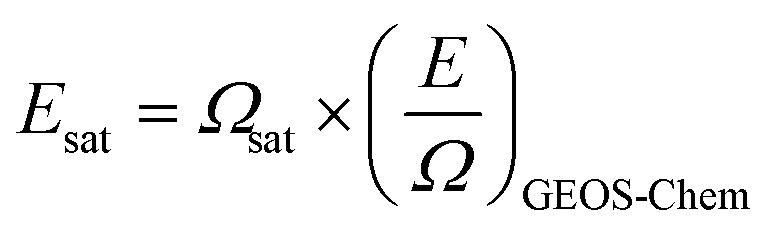
*Ω*_sat_ is monthly mean TROPOMI NO_2_ or IASI NH_3_ column densities and (*E*/*Ω*)_GEOS-Chem_ is the modelled ratio of 24 h monthly total NO_*x*_ or NH_3_ emissions to column densities of NO_2_ or NH_3_ averaged during the satellite overpass (Section 2.4). This approach attributes all the discrepancy between the satellite observations and model to biomass burning, so eqn (3) is only used for gridboxes with >50% contribution of biomass burning to total emissions, according to GEOS-Chem (Section 2.3). We use modelled emissions and columns driven with inventories that yield best agreement with TROPOMI for NO_*x*_ and with IASI for NH_3_.

Interpretation of the spatial distribution of the emissions calculated with eqn (3) is aided by the global 0.5° pyrome classification dataset^59^ archived by the Archibald Ecology Laboratory (https://archibaldlab.weebly.com/datasets.html, last accessed 25 February 2025) (Fig. S1†). Pyromes are classed by frequency, intensity, and size from Bayesian clustering informed by datasets of active fires, burned area, fire radiative power, fire season, ecoregions, and variables of climate and human influence.

## 3. Results

### 3.1 Bottom-up biomass burning NO_*x*_ and NH_3_ emissions

**Fig. 1:**
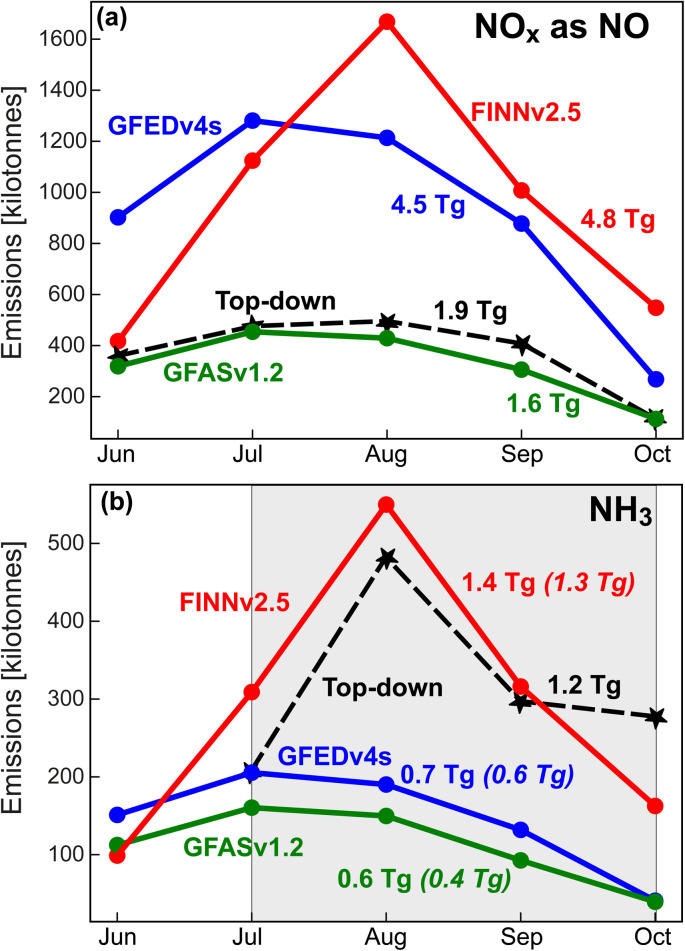
Comparison of monthly biomass burning NO_*x*_ and NH_3_ emissions. Panels compare subtropical southern Africa biomass burning emissions of NO_*x*_ (as NO) (a) and NH_3_ (b) in 2019 from the inventories GFEDv4s (blue), FINNv2.5 (red), and GFASv1.2 (green) and from our top-down estimate (black; Section 3.4). Inset regular font values are inventory and top-down totals for June–October, except for top-down NH_3_ that is for July–October (time period shaded grey). Italicized values in parentheses are bottom-up NH_3_ for July–October only.

Fig. 1 compares monthly June–October NO_*x*_ and NH_3_ emissions from the three inventories. NO_*x*_ emissions totals are similar for GFEDv4s (4.5 Tg NO) and FINNv2.5 (4.8 Tg NO) and about 3-times less for GFASv1.2 (1.6 Tg NO). NH_3_ emissions totals are similar for GFEDv4s (0.72 Tg) and GFASv1.2 (0.55 Tg) and at least double for FINNv2.5 at 1.4 Tg. Emissions peak in July according to GFEDv4s and GFASv1.2, and in August for FINNv2.5. Month-to-month variability is similar for GFEDv4s and GFASv1.2. FINNv2.5 exhibits distinct and greater seasonal variability than the other two inventories. FINNv2.5 emissions increase from similar emissions to GFASv1.2 in June to 4-times more NO_*x*_ and 6-times more NH_3_ in August and sustaining 2- to 5-times more NO_*x*_ and 4-times more NH_3_ than the other inventories in October.

Both dry matter burned and the choice of emission factors (Table 1) contribute to systematic differences in NO_*x*_ emissions between GFEDv4s and GFASv2.1. GFEDv4s uses a savanna emission factor (3.9 g NO_*x*_ as NO (kg DMB)^−1^) that is almost double GFASv1.2 (2.1 g NO_*x*_ as NO (kg DMB)^−1^). The effect of greater burned area in GFEDv4s is evident from differences in NH_3_ emissions. The ∼40% greater emission factors for GFASv1.2 (0.74 g (kg DMB)^−1^) than GFEDv4s (0.52 g (kg DMB)^−1^) is offset by more dry matter burned in GFEDv4s. FINNv2.5 uses the same NO_*x*_ emission factor as GFEDv4s for savannas, and the chapparal vegetation type emission factor of 3.65 g NO_*x*_ as NO (kg DMB)^−1^ for woody savannas that is similar to that for savannas, so differences between these two inventories is mostly due to estimated dry matter burned and more intense equatorward tropical forest fires in FINNv2.5. The forest fire emission factor in FINNv2.5 (2.5 g NO_*x*_ as NO (kg DMB)^−1^) is less than the savanna emission factors, but the fuel consumed is far greater in FINNv2.5. FINNv2.5 burn fractions (*α* in eqn (2)) of 0.9 for savannas and 0.3 for tropical forests^40^ and southern Africa fuel loads (*B* in eqn (2)) of 411 g m^−2^ for savannas and 25 295 g m^−2^ for tropical forests^36^ amounts to ∼20-times more fuel consumed for tropical forests than savannas.

Distinct FINNv2.5 NH_3_ emissions is in part because 20–30% of gridboxes with active fires are classified as woody savanna that has an emission factor (1.2 g (kg DMB)^−1^) that is more than double that for savannas (Table 1). The proportion of woody savanna gridboxes in FINNv2.5 increases from ∼20% in June to ∼30% in August and declines back to ∼20% in October. Sustained October emissions in FINNv2.5 is because of widespread emissions in Angola and southwest Zambia that are either absent or far less intense in the other inventories.

Addition of VIIRS in FINNv2.5 increases total regional June–October NH_3_ and NO_*x*_ emissions by ∼20% relative to emissions obtained with MODIS only. The effect on spatial coverage of emissions is small. Emissions using both VIIRS and MODIS sensors results in >600 000 more 0.1° daily gridboxes with emissions than the emissions product that uses MODIS only. Though this is a cumulative area of >700 000 km^2^, the additional VIIRS gridboxes only amounts to 143 kt more NO_*x*_ as NO or just 3% of the Fig. 1(a) total. The increase in NH_3_ emissions for these additional gridboxes is slightly more, at 8% (114 kt) of the Fig. 1(b) total.

In June–September, biomass burning dominates total boundary layer (<2 km) NO_*x*_ emissions in subtropical southern Africa, according to GEOS-Chem using GFEDv4s (monthly emissions range is 79–87% of total NO_*x*_ emissions), FINNv2.5 (72–87%), and GFASv1.2 (58–70%). The other prominent source is soil NO_*x*_, totalling 110–190 kt NO or ∼10–30% of boundary layer NO_*x*_ emissions. By October, soil NO_*x*_ emissions of 200 kt NO are 86 kt more than GFASv1.2, 71 kt less than GFEDv4s, and less than half the 550 kt NO from FINNv2.5. Monthly anthropogenic NO_*x*_, mostly from combustion of vehicular fuels and domestic burning of biomass and charcoal,^55,60,61^ is a much smaller NO_*x*_ source than biomass burning and soils at 41–43 kt NO in June–October. Lightning is another prominent NO_*x*_ source in the region, but most (>95%) is emitted above the boundary layer, increasing from 28 kt NO in June to 140 kt NO in October with the transition to the rainy season.

For NH_3_, the biomass burning contribution to total emissions is similar in June–September for GFEDv4s (40–52%) and GFASv1.2 (32–46%), but declines to 17–18% in October. For FINNv2.5, the contribution increases from 35% in June to 46–74% in July–October. The other major NH_3_ source is anthropogenic, totalling 170–180 kt in each month from activities such as agriculture and charcoal production in rural areas and from vehicles and domestic burning of waste, biomass, and charcoal in urban areas.^55,60,61^ More than half (∼53%) of this anthropogenic NH_3_ is concentrated north of 5°S. Monthly natural NH_3_ emissions total 19–21 kt.

A new GFED version (GFEDv5) has been developed that is undergoing quality checks and validation before final release (https://www.globalfiredata.org/data.html; last accessed 12 May 2025). GFEDv5 updates emission factors to the latest Binte Shahid *et al.*,^24^ resulting in emissions for June–October that are 1.2 Tg more than GFEDv4s for NO_*x*_ at 5.7 Tg NO and ∼0.5 Tg more than GFEDv4s for NH_3_ at 1.2 Tg. The NH_3_ emissions seasonality shifts to a more pronounced August peak of 0.35 Tg for GFEDv5 compared to <0.2 Tg for GFEDv4s (Fig. 1(b)).

### 3.2 Evaluation of bottom-up NO_*x*_ emissions with TROPOMI NO_2_ and implications for ozone and PAN

**Fig. 2:**
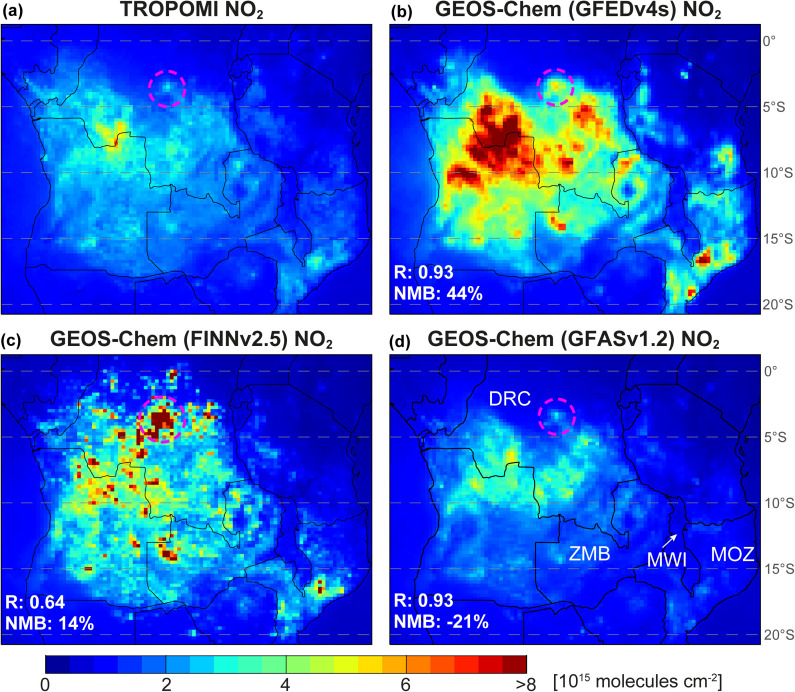
Observed *vs.* modelled June–October mean tropospheric column densities of NO_2_. Panels are gridded (0.25° × 0.3125°) 2019 TROPOMI observations (a) and coincident GEOS-Chem using GFEDv4s (b), FINNv2.5 (c), and GFASv1.2 (d) all with TROPOMI averaging kernels applied (Section 2.4; Text S1†). Inset values in (b)–(d) are Pearson's correlation coefficients (*R*) and the model normalized mean bias (NMB) for the domain plotted. Countries discussed in Section 3.2 are indicated in (d) (DRC = Democratic Republic of the Congo, MOZ = Mozambique, MWI = Malawi, ZMB = Zambia). Pink dashed circles in all panels collocate the NO_2_ hotspot from tropical forest fires discussed in the text.

Fig. 2 compares TROPOMI and GEOS-Chem tropospheric NO_2_ column densities averaged over June–October. Modelled NO_2_ obtained with GFEDv4s and GFASv1.2 is more spatially consistent with TROPOMI (*R* = 0.93 for both) than FINNv2.5 (*R* = 0.64). The correlation in individual months exceeds 0.82 for GFEDv4s and GFASv1.2 and ranges from *R* = 0.42 in July to *R* = 0.75 in October for FINNv2.5. GEOS-Chem seasonal domain mean NO_2_ is most consistent with TROPOMI using FINNv2.5 (NMB = 14%) and GFASv1.2 (NMB = −21%) compared to GFEDv4s (NMB = 44%). Due to large monthly variability in FINNv2.5 and GFEDv4s emissions (Fig. 1(a)), NMBs range from 2% in September to 50% in August for FINNv2.5 and from 1% in October to 75% in July for GFEDv4s. Those for GFASv1.2 are least variable at −14% in July to −28% in September. In June–September, FINNv2.5 NO_*x*_ emissions in the northern portion of the domain (pink dashed circles in Fig. 2) far exceed the other inventories. The northly extent of emissions in FINNv2.5 is in both the combined VIIRS and MODIS and the MODIS-only product and is because landcover there is tropical forest that, according to FINNv2.5, has 20-times greater fuel consumption than savannas.^36^

The model overestimate in the integrated tropospheric column densities of NO_2_ using GFEDv4s (Fig. 2(b)) is also apparent in comparisons of GEOS-Chem to discrete vertical profiles of free tropospheric NO_2_ obtained by cloud-slicing total columns of TROPOMI NO_2_. In that comparison, the model driven with GFEDv4s is more than double the June–August mean cloud-sliced NO_2_ mixing ratios at 800–600 hPa (∼2–4 km) over southern Africa.^62^

The averaging kernels applied to GEOS-Chem (Section 2.4; Text S1†) alter most monthly mean model gridboxes by ∼10%, with the exception of a few gridboxes in the DRC with larger enhancements in NO_2_ (>5 × 10^15^ molecules cm^−2^) due to fires. These decline by 1–2 × 10^15^ molecules cm^−2^ or 20–30%, due to the relatively poor sensitivity of TROPOMI to the lower troposphere.^63^ The largest decline of 5–10 × 10^15^ molecules cm^−2^ (25–65% decrease) is in August for NO_2_ > 15 × 10^15^ molecules cm^−2^ in central DRC obtained with GEOS-Chem using FINNv2.5.

A striking feature in Fig. 2(a)–(c) is the much lower NO_2_ concentrations in Malawi than its neighbours Zambia and Mozambique. Malawi is amongst the least fire-prone countries in southern Africa, whereas its neighbours Zambia and Mozambique are amongst the most, based on 8 years of burned area data.^64^ Malawi's mostly rural population density is ∼220 people km^−2^, far more than its neighbours (<50 people km^−2^) (https://data.worldbank.org/indicator/EN.POP.DNST, last accessed 5 March 2025). There is a steep, exponential decline in fire size with population density.^12^ A greater population density increases fire occurrence, but it also fragments the land, preventing fires that reach Malawi from propagating.^12,64^

Differences in the NO_*x*_ emissions in Fig. 1(a) affect chemical production of ozone and PAN. According to GEOS-Chem, total chemical production of boundary layer ozone (O_3_) in June–October is 20 Tg using GFASv1.2 that emits less NO_*x*_ than the other inventories (Fig. 1(a)). Ozone production with the other inventories is 11 Tg more than GFASv1.2 using GFEDv4s and 26 Tg more using FINNv2.5. Even though GFEDv4s and FINNv2.5 yield greater ozone production than GFASv1.2, the ozone production efficiency (OPE) for GFASv1.2 exceeds the other inventories. OPE for GFASv1.2 is 13 Tg O_3_ (Tg NO)^−1^ compared to 6.9 Tg O_3_ (Tg NO)^−1^ for GFEDv4s and 9.6 Tg O_3_ (Tg NO)^−1^ for FINNv2.5. Far more of the other ozone precursors, CO and non-methane volatile organic compounds (NMVOCs), in FINNv2.5 cause the greater OPE than GFEDv4s. FINNv2.5 emissions total 108 Tg CO and 13 Tg C for 21 NMVOCs compared to 82 Tg CO and 2.0 Tg C for 13 NMVOCs for GFEDv4s and 49 Tg CO and 0.82 Tg C for 12 NMVOCs for GFASv1.2. If all NO_*x*_ in FINNv2.5 is emitted as NO rather than mostly NO_2_ (Section 2.1; Table 1), the OPE declines to 9.1 Tg O_3_ (Tg NO)^−1^, as there is more NO to react directly with ozone.

Boundary layer PAN is also affected by differences in NO_*x*_ emissions. PAN production with FINNv2.5 totals 3.6 Tg for June–October. This far exceeds the other inventories by 2.7 Tg for GFEDv4s and 3.1 Tg for GFASv1.2. In addition to differences in NO_*x*_ emissions, FINNv2.5 also includes NMVOCs with large PAN yields that are absent in the other inventories. Specifically, methyl glyoxal, methyl vinyl ketone, methacrolein, and hydroxyacetone.^65^ PAN production efficiencies are 0.8 Tg PAN (Tg NO)^−1^ for FINNv2.5, 0.2 Tg PAN (Tg NO)^−1^ for GFEDv4s and 0.3 Tg PAN (Tg NO)^−1^ for GFASv1.2. If all FINNv2.5 NO_*x*_ is emitted as NO, PAN production only declines by 0.1 Tg.

### 3.3 Evaluation of bottom-up NH_3_ emissions with IASI

**Fig. 3:**
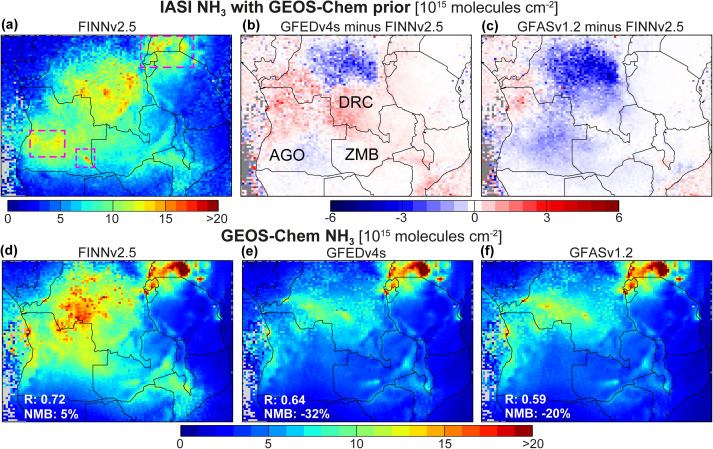
Comparison of IASI and GEOS-Chem July–October mean NH_3_. All gridded (0.25° × 0.3125°) maps are for 2019. Panels are IASI NH_3_ retrieved with GEOS-Chem priors obtained using FINNv2.5 (a), the difference between (a) and IASI reprocessed with GEOS-Chem using GFEDv4s (b) and GFASv1.2 (c), and the GEOS-Chem IASI NH_3_ columns obtained with FINNv2.5 (d), GFEDv4s (e) and GFASv1.2 (f). Grey grids mostly over the Atlantic Ocean lack at least 10 IASI pixels in all months (Text S2†). Values in (d)–(f) are Pearson's correlation coefficients (*R*) between IASI and the model and the model normalized mean bias (NMB) for the domain plotted. Countries discussed in Section 3.3 are indicated in (b) (AGO = Angola, DRC = Democratic Republic of the Congo, ZMB = Zambia). Pink dashed boxes in (a) identify features in Angola to the south and the Ukerewe basin in the north discussed in the text.

Fig. 3 compares NH_3_ columns from the reprocessed IASI data and from GEOS-Chem for July–October. Even though we use IASI NH_3_ from both MetOp-A and -B, data density is 20 to 24 times less than TROPOMI, due to the coarser IASI pixel resolution (Section 2.1). All three reprocessed products are very similar to each other, so differences relative to FINNv2.5 are shown for GFEDv4s (Fig. 3(b)) and GFASv1.2 (Fig. 3(c)). The spatial correlation (*R*) between products for individual months exceeds 0.95 and the relative difference in domain mean IASI NH_3_ is <10%. More NH_3_ emissions from FINNv2.5 compared to the other inventories (Fig. 1(b)) causes greater column densities in the DRC in the northcentral portion of the model domain. These are about 3–4 × 10^15^ molecules cm^−2^ more in FINNv2.5, but the NH_3_ columns there are still less prominent than the NH_3_ due south in southern DRC. The model profile shape rather than magnitude is used in the retrieval, so there is limited influence of the prior on the spatial distribution of NH_3_, as is evident in the distinct spatial distribution of NH_3_ for the reprocessed IASI columns in Fig. 3(a) and the prior (Fig. 3(d)).

Replacing the default prior with GEOS-Chem leads to a systematic, extensive decrease in NH_3_ column densities (Fig. S2†), as more NH_3_ is distributed to higher altitudes in GEOS-Chem than the default terrestrial *a priori* profile. This was also the case for IASI PAN reprocessed with GEOS-Chem priors.^58^ Specific NH_3_ enhancements in the provided product that decrease on reprocessing include a persistent NH_3_ enhancement along the west coast of Angola of 17–30 × 10^15^ molecules cm^−2^ that declines to <15 × 10^15^ molecules cm^−2^ with GEOS-Chem, and a decrease in the intensity of NH_3_ in the Ukerewe (Lake Victoria) basin. The Ukerewe basin enhancement still occurs in the reprocessed product (pink dashed box in Fig. 3(a)) and is likely associated with anthropogenic activity, as this is one of the most densely populated, predominantly rural, regions in Africa.^66,67^ The reprocessed NH_3_ also has smoother spatial gradients than the provided product, such as along coastlines where the provided product *a priori* transitions from a fixed ocean to a fixed land vertical NH_3_ profile. Fewer retrieved pixels are also removed in the postfilter step (Section 2.4) with GEOS-Chem as prior.

June is not in Fig. 3, as the spatial correlation is poor for all inventories (*R* < 0.5). The correlation improves to *R* = 0.62–0.83 in July–September and, in October, is stronger for FINNv2.5 (*R* = 0.70) than the other inventories (*R* = 0.38 for both). The major biomass burning NH_3_ enhancement in the reprocessed IASI NH_3_ in June that GEOS-Chem does not reproduce is fires in Angola. These include a widespread enhancement in NH_3_ south of central Angola and a smaller, more intense well-defined plume along the border with Zambia, also apparent in July–October (Fig. 3(a)). This feature is absent in non-biomass burning months (Fig. S3†) and TROPOMI NO_2_ is only marginally enhanced (<2.5 × 10^15^ molecules cm^−2^) in June over southern Angola, suggestive this is smouldering burning undetected by MODIS or VIIRS. Predominance of smouldering fires in June is consistent with the low combustion efficiency estimated by Fang *et al.*^22^ using satellite observations of CO and an assimilated CO_2_ product. They attributed low combustion efficiency in June to relatively high fuel moisture content at the end of the rainy season.

Model NMBs for individual months are consistently biased high (NMB = 13–45%) in July and almost all biased low in August–October (NMB of −2% to −63%), except for FINNv2.5 that has a positive bias of 12% in August when its emissions far exceed the other inventories (Fig. 1(b)).

### 3.4 Top-down biomass burning NO_*x*_ and NH_3_ emissions

We infer 24 h monthly NO_*x*_ and NH_3_ emissions using eqn (3) (Section 2.5) and modelled emissions and columns driven with GFASv1.2 for NO_*x*_, as it is most consistent with TROPOMI (Fig. 2), and FINNv2.5 for NH_3_ for the same reason (Fig. 3). Emissions are also only estimated for months when GEOS-Chem and the satellite data are spatially correlated. These are June–October for NO_*x*_ (*R* = 0.85–0.94) and July–October for NH_3_ (*R* = 0.68–0.81). According to the bottom-up inventories, model gridboxes with >50% biomass burning contribution to total emissions account for 93% of total biomass burning emissions for NO_*x*_ and 94% for NH_3_.

**Fig. 4:**
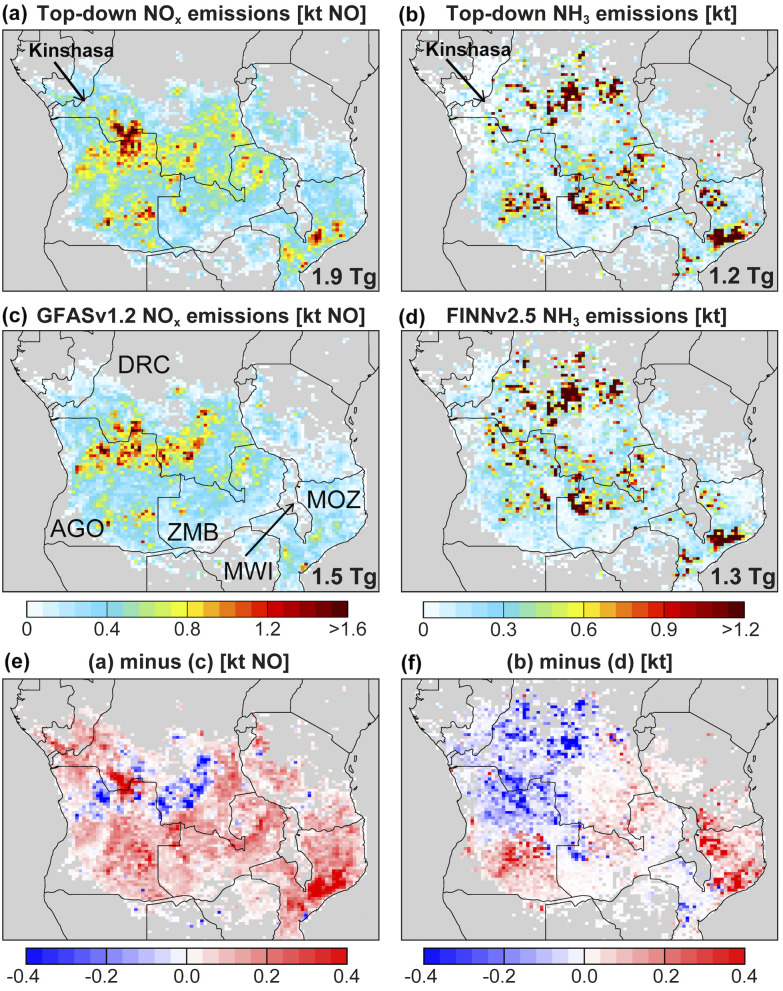
Comparison of top-down and bottom-up reactive nitrogen emissions. Panels are top-down NO_*x*_ (a) and NH_3_ (b), bottom-up GFASv1.2 NO_*x*_ (c) and FINNv2.5 NH_3_ (d), and the difference between top-down and bottom-up NO_*x*_ (e) and NH_3_ (f). Grey gridboxes have <50% biomass burning contribution to total emissions in all months, according to GEOS-Chem (Section 2.3). Arrows in (a) and (b) point to grey omitted gridboxes in and neighbouring Kinshasa discussed in Section 4. Inset values in (a)–(d) are emissions totals for NO_*x*_ in June–October and NH_3_ in July–October. Top-down emissions totals for individual months are in Fig. 1. Countries discussed in Section 3.4 are indicated in (c) (AGO = Angola, DRC = Democratic Republic of the Congo, MOZ = Mozambique, MWI = Malawi, ZMB = Zambia).

Fig. 4 compares maps of collocated multi-month total top-down and bottom-up emissions of NO_*x*_ and NH_3_. Top-down June–October NO_*x*_ emissions total 1.9 Tg compared to 1.5 Tg from GFASv1.2, due to widespread increases in emissions almost everywhere except northern Angola and southeast DRC. Top-down July–October NH_3_ emissions total 1.2 Tg. This is only 27 kt less than FINNv2.5, as regional decline in top-down emissions in most of the west is balanced by increases in the east and in southern Angola. NO_*x*_ emissions across the domain are distributed normally (mean = 0.39 kt, median = 0.36 kt), whereas NH_3_ has a long tail distribution (mean = 0.27 kt, median = 0.14 kt). Bottom-up and top-down emissions are very spatially consistent (*R* = 0.88 for NO_*x*_, *R* = 0.89 for NH_3_), as expected from selection of these inventories from comparison to TROPOMI and IASI (Fig. 2 and 3).

NO_*x*_ emissions in northern Angola and southern DRC collocate with the yellow boomerang-shaped pyrome (Fig. S1†) of frequent, intense and large (FIL) fires that preferentially undergo flaming combustion. This is similarly the case for NO_*x*_ emissions in northeast Zambia and southeast Angola. The NO_*x*_ hotspot along the Angola/DRC border and also coincident with FIL fires is more pronounced in the top-down than the bottom-up emissions (Fig. 4(a)*versus*(c)), as expected from the relatively large TROPOMI tropospheric NO_2_ column abundances in this location (Fig. 2(a)).

Many of the intense NH_3_ emissions in Fig. 4(b) are spatially distinct from the intense NO_*x*_ emissions, except for those in Mozambique due south of Malawi. The correlation between top-down NO_*x*_ and NH_3_ emissions in individual months is <0.4. Similarly, GFASv1.2 NO_*x*_ and FINNv2.5 NH_3_ emissions are weakly correlated (also *R* < 0.4). The spatial consistency between NO_*x*_ and NH_3_ emissions is much greater for each inventory (*R* > 0.99 for GFASv1.2, *R* > 0.87 for FINNv2.5).

Exclusive NH_3_ emissions in the DRC coincide with fires classified as cool and small (Fig. S1†), so are more prone to smouldering, favouring formation of NH_3_ over NO_*x*_. These cool and small fires decline in frequency from occurring often to intermediate to rare with northerly extent. Both top-down and bottom-up NH_3_ emissions extend further north into the Congolese forest than the pyrome regime map, likely because of encroachment of fires since the pyrome classification data record end date of 2010. Multiple independent studies corroborate a steep, statistically significant increase in fires at the southern edge of the Congolese forest^68–70^ attributed to warmer, drier conditions.^68^ Over the decade from the end of the pyrome classification time period (2010) to the observation record used here (2019), fires have increased by >50 active fires per 0.25° (∼28 km resolution) gridbox, based on trends in MODIS active fires.^69^

Monthly top-down emissions totals are also shown in Fig. 1 for comparison to the bottom-up values. Top-down NO_*x*_ emissions shift the emissions peak from July in GFASv1.2 to August, though the top-down emissions estimate for July and August only differs by 19 kt. The top-down NO_*x*_ emissions suggest fires in subtropical southern Africa produce ∼25 Tg boundary layer ozone, based on an OPE of 13 Tg O_3_ (Tg NO)^−1^ obtained with GEOS-Chem using GFASv1.2 NO_*x*_ emissions (Section 3.2) and assuming linearity across the 0.4 Tg difference between the top-down and bottom-up NO_*x*_ emissions. Top-down NH_3_ emissions are less than FINNv2.5 in all months (by 79 kt in July, 59 kt in August, and 9 kt in September), except October that is 120 kt more than FINNv2.5. Emissions peak in August for both estimates, though the top-down peak is less pronounced than FINNv2.5.

If GFEDv4s or FINNv2.5 instead of GFASv1.2 is used to estimate NO_*x*_ emissions, June–October totals are 2.2 Tg NO using GFEDv4s and 2.4 Tg NO using FINNv2.5 (Fig. S4(a)†). This is only 0.3–0.5 Tg more than the top-down emissions derived with GFASv1.2 and far more consistent than the 3.2 Tg NO spread in bottom-up emissions (Fig. 1(a)). All top-down estimates also peak in August and there is a substantial (∼1 Tg) decrease in the prominence of the FINNv2.5 August peak. Remaining differences in top-down NO_*x*_ emissions likely result from errors in free tropospheric NO_2_ (ref. 71) where TROPOMI is most sensitive to NO_2_.

If, for NH_3_, GFEDv4s or GFASv1.2 is used to calculate top-down emissions, July–October totals are 0.9 Tg using GFEDv4s and 0.6 Tg using GFASv1.2 (Fig. S4(b)†). The values converge on a 0.6 Tg difference for top-down compared to 0.9 Tg difference for bottom-up. A large portion (∼0.2 Tg) of this spread is because many of the northerly equatorward forest fire emitting gridboxes in FINNv2.5 are absent in GFEDv4s and GFASv1.2, so the GEOS-Chem term in eqn (3) is zero for these gridboxes. Top-down emissions using GFEDv4s and GFASv1.2 shift the peak from July to August, but neither is as pronounced as FINNv2.5 (Fig. S4(b)†).

### 3.5 Error analysis of the top-down emissions

Potential sources of uncertainty in the top-down emissions include the satellite observations, the GEOS-Chem term in eqn (3), and GEOS-Chem inventories used to identify gridboxes with >50% contribution from biomass burning.

According to past error estimates for TROPOMI NO_2_, the error is typically ∼30% and is dominated by the air mass factor used to convert slant columns to vertical column densities.^45^ The IASI NH_3_ relative error for the version 4 product we use is 19–36%.^33^ These error estimates are for individual observations, so the random component decreases substantially by averaging over multiple months.

We quantify GEOS-Chem error contributions from emissions perturbation simulations. For the GEOS-Chem term in eqn (3), we assess the percent change in top-down emissions due to a perturbation in biomass burning emissions informed by differences between top-down and the selected bottom-up inventories in Fig. 1. Perturbation simulations are for August when emissions in NH_3_ and NO_*x*_ peak. GFASv1.2 NO_*x*_ emissions are increased by 20% and FINNv2.5 NH_3_ emissions are reduced by 12%. The domain mean change in top-down emissions for the same gridboxes shown in Fig. 4 is a ∼3% increase in NO_*x*_ emissions and a ∼2% decline in NH_3_ emissions. The small change in emissions is because the perturbation in emissions causes a near-equal response in the column, as has been reported previously for top-down estimate of UK agricultural NH_3_ emissions.^50^

Bottom-up inventories of anthropogenic emissions are very uncertain and could impart errors in identifying gridboxes with >50% contribution from biomass burning. For NO_*x*_, we test sensitivity to this by doubling anthropogenic NO_*x*_ emissions, prompted by the suggestion that these are underestimated in urban areas from a study that evaluated bottom-up emissions against single point measurements in 3 urban areas in Angola and 1 urban area in Zambia.^72^ For NH_3_, informed by our own comparison of IASI and GEOS-Chem (Fig. 3), we halve anthropogenic NH_3_ emissions, as the model overestimates NH_3_ column densities over the densely populated Ukerewe basin (Section 3.3). The resultant biomass burning season (June–October for NO_*x*_, July–October for NH_3_) emissions are only 1% (25 kt NO) less than in Fig. 4(a) for NO_*x*_ and 3% (34 kt) more than Fig. 4(b) for NH_3_. The limited sensitivity to biases in anthropogenic emissions is because these emissions are in populated areas where fire propagation is supressed.^12^

Conservatively, relative error contributions for NO_*x*_ emissions are 0.3 for TROPOMI, 0.03 for the GEOS-Chem term in eqn (3), and 0.01 for anthropogenic NO_*x*_ emissions. Adding these in quadrature yields total NO_*x*_ emissions of 1.9 ± 0.6 Tg. Similarly, for NH_3_, contributions are at most 0.36 for IASI, 0.02 for the GEOS-Chem term in eqn (3), and 0.03 for anthropogenic NH_3_ emissions. Domain total NH_3_ emissions are then 1.2 ± 0.4 Tg.

## 4. Discussion

None of the inventories include primary sulfate and nitrate aerosol emissions. As a result, GEOS-Chem may overestimate NH_3_ columns, due to an underestimate in partitioning of semi-volatile NH_3_ to these acidic aerosols to form ammonium aerosol. We test sensitivity of modelled NH_3_ to inclusion of primary sulfate and nitrate emissions by adding these to FINNv2.5, given its greater consistency with IASI (Fig. 3(e)). For simplicity, we allocate tropical forest sulfate and nitrate emission factors to fires north of 5°S and west of 30°E and savanna sulfate and nitrate emission factors to all other fires. The emission factors we use (per kg DMB) are 130 mg sulfate and 110 mg nitrate for tropical forests and 18 mg sulfate and 16 mg nitrate for savannas and woody savannas.^25^ No emission factors are given for the chapparal landcover type used in FINNv2.5 for woody savannas (Section 3.1). The effect on the modelled NH_3_ columns is near-negligible. With primary sulfate and nitrate, the model correlation is unchanged and the model NMB is only 1 percentage point less than in Fig. 3(d).

GFASv1.2 is the only inventory with recommended injection heights (Section 2.1). Emissions injected above the boundary layer would affect the comparisons in Fig. 2 and 3, due to variability in vertical sensitivity of the two instruments (Section 2.4). The GFASv1.2 injection heights in subtropical southern Africa typically extend to ∼3 km, but most (∼80%) emissions are released to the lowest 5 layers of the model, reaching 750–850 m above ground level. This is well within the daytime boundary layer when most biomass is burned (Section 2.1). Turning this injection height feature off in GEOS-Chem has no effect on the comparison statistics for NO_2_ in Fig. 2(d) and NH_3_ in Fig. 3(d), as GEOS-Chem immediately mixes surface layer emissions throughout the boundary layer.^73^

There are more complex and computationally intensive approaches than eqn (3) to infer emissions from satellite observations. Some explicitly account for effects like non-linear chemistry and displacement of the observed trace gas from the emission gridbox or so-called smearing.^30,74^ Such approaches are suitable for static perennial or seasonal sources, like anthropogenic or biogenic (vegetation) emissions, but are not practical for episodic biomass burning emissions. Another option is iteration that would account for non-linear chemistry and model errors in the amount and vertical distribution of free tropospheric NO_2_. The top-down emissions obtained in our study would be embedded in the model or used to scale the prior emissions to simulate top-down-informed columns that would then be used to obtain new top-down emissions. This process would be repeated until a pre-defined convergence criterion is met,^75^ but such an approach is computationally costly. Another inversion approach is application of wind rotation and a plume fitting model to TROPOMI NO_2_ to estimate NO_*x*_ emissions of individual fire plumes.^76^ This method has been successfully applied to individual fires in Africa using daily TROPOMI observations, but it only yields top-down emissions for select isolated plumes with a well-defined Gaussian shape on wind rotation.^77^ Even so, the plume NO_*x*_ emissions that were derived exhibit a strong linear relationship with fire radiative power^77^ that we also find is a suitable explanatory variable for NO_*x*_ emissions (Section 3.2).

Our top-down emissions would ideally be validated by simulating GEOS-Chem with these top-down emissions and comparing modelled concentrations to independent ground-based observations of NO_*x*_ and NH_3_. The long-term International Network to Study Deposition and Atmospheric composition in Africa (INDAAF) designed to monitor dry and wet deposition includes trace gas surface concentration measurements of NH_3_ and NO_2_, but all are located outside the latitude band considered here.^78^ The recent intensive (January 2019) Methane Observations and Yearly Assessments (MOYA) aircraft campaign sampled biomass burning plumes over Uganda, but these were for the northern hemisphere burning season and limited to CO and long-lived greenhouse gases.^79^ There are routine commercial aircraft observations from the In-service Aircraft for a Global Observing System (IAGOS) programme, but these flights sample the vertical distribution of the troposphere at airports dominated by anthropogenic pollution or influenced by long-range transported biomass burning plumes.^7^

Validation of the satellite observations for conditions relevant to this work is also not feasible. There was a ground-based Multi-Axis Differential Optical Absorption Spectroscopy (MAX-DOAS) instrument measuring vertical column densities of NO_2_ in Burundi for assessment of space-based tropospheric columns of NO_2_,^80^ but it ceased operating before TROPOMI launched and would anyway have been mostly influenced by anthropogenic emissions from the densely populated Ukerewe basin. A MAX-DOAS instrument has been operating in the fast-growing capital city of the DRC, Kinshasa, since 2019,^81^ but data over this city are excluded in the top-down inference of emissions (arrows in Fig. 4(a) and (b)), as emissions are mostly from non-biomass burning sources. Optimum locations of ground-based instruments to validate satellite observations of biomass burning NO_2_ and NH_3_ are national parks where burning is intense and propagates over large areas.^12^

Our results suggest that the most suitable approach to estimate byproducts of flaming fires is to use either burned area or fire radiative power products with a savanna NO_*x*_ emission factor of 2.1 g (kg DMB)^−1^. Though this NO_*x*_ emission factor is unpublished, it is similar to the mean value of 2.4 g (kg DMB)^−1^ reported by Andreae.^26^ Other byproducts that would similarly be produced in relative abundance with these fires include black carbon and carbon dioxide (CO_2_).

For smouldering fire emissions, the most suitable approach is application of landscape-specific fuel loadings and burning completeness fractions to active fires and NH_3_ emission factors that distinguish landcover by the relative coverage of woody vegetation, as in FINNv2.5. Co-emitted smouldering fire byproducts include CO, organic aerosols, methane, and NMVOCs. The distinct August peak in NH_3_ emissions in Fig. 1(b) is corroborated by top-down estimates of CO emissions for southern Africa from inversion of satellite observations of CO^21^ and from bottom-up emissions estimates using the very high spatial resolution (20 m) Sentinel-2 instrument for enhanced detection of small fires.^82^ The top-down CO emissions from Zheng *et al.*^21^ are 1.5 to 2 times more than CO from GFASv1.2 and GFEDv4s in August–October. Combustion efficiency, determined as the ratio of CO_2_ to the sum of CO and CO_2_, also declines from ∼0.93 in May–July to 0.84–0.87 in August–October due to an increase in fuel moisture content as the region transitions to the rainy season.^21^

The GFASv1.2 spatial consistency with TROPOMI NO_2_ would likely also occur with the NASA Quick Fire Emissions Dataset (QFED) inventory that too is generated with fire radiative power (https://gmao.gsfc.nasa.gov/pubs/docs/Darmenov796.pdf). The next version of GFED (v5) would worsen the discrepancy with TROPOMI NO_2_ in Fig. 2(b) and with the TROPOMI-derived NO_*x*_ emissions. GFEDv5 NH_3_ emissions reproduce the IASI-derived domain total emissions (both 1.2 Tg) and would better match the top-down seasonality in Fig. 1(b) than GFEDv4s. Consistency with the spatial distribution of IASI NH_3_ may remain an issue, as the distinct enhancement in NH_3_ emissions in the southern edge of the Congolese forest in Fig. 4(b) is absent in GFEDv5.

## 5. Conclusions

We determined reactive nitrogen emissions of NO_*x*_ and NH_3_ for the 2019 burning season in subtropical southern Africa using the GEOS-Chem model driven with three distinct biomass burning inventories (FINNv2.5, GFEDv4s, GFASv1.2) and satellite observations of NO_2_ from TROPOMI and NH_3_ from IASI. Mass balance top-down emissions estimates used GEOS-Chem driven with inventories yielding column density abundances with greatest spatial and regional mean consistency with TROPOMI (GFASv1.2) and with IASI (FINNv2.5).

Our top-down estimated biomass burning emissions total 1.9 ± 0.6 Tg NO_*x*_ as NO for June–October and 1.2 ± 0.4 Tg NH_3_ for July–October. The satellite observations make the largest contribution to uncertainties in the emissions estimates. June is excluded for NH_3_, due to poor agreement of GEOS-Chem with IASI using all three inventories. The model does not reproduce the IASI NH_3_ enhancements in Angola that may be due to smouldering fires at the start of the burning season. The IASI observations suggest then that the burning season initiates in the southwest, upending current understanding that burning begins in the north and propagates south.

We find with GEOS-Chem sensitivity simulations that our top-down emissions estimates of NO_*x*_ and NH_3_ are unaffected by including plume injection height, due to the relatively low altitude of fire plumes in this region, and that emissions of NH_3_ are unchanged by inclusion of primary emissions of acidic sulfate and nitrate aerosols that promote partitioning of semi-volatile NH_3_ to aerosols.

We additionally derive a top-down informed June–October ozone production efficiency (OPE) of 13 Tg O_3_ (Tg NO)^−1^. Far greater GFEDv4s (4.5 Tg NO) and FINNv2.5 (4.8 Tg NO) NO_*x*_ emissions than the top-down estimate decreases the OPE to 7–10 Tg O_3_ (Tg NO)^−1^ due to transition to a far less NO_*x*_-sensitive O_3_ production regime. PAN production is greatest with FINNv2.5, due to inclusion of high-yielding PAN precursor NMVOCs.

All inventories collocate NO_*x*_ and NH_3_ emissions, whereas top-down estimates suggest these are distinct for almost all fires, supportive of a hybrid bottom-up approach. Such an inventory could apply landscape specific fuel loads and combustion completeness to active fires for smouldering emissions and burned area or fire radiative power data for flaming emissions. Still, the June southern Angola enhancement in NH_3_ would be absent in this hybrid approach.

## Author contributions

Study concept, formal analysis, investigation, methodology, and original draft by EAM, data provision and guidance on use by MVD and LC for IASI, CW for FINNv2.5, GvdW for GFEDv5, and KM for GFASv1.2 implementation in GEOS-Chem. All authors contributed to editing and reviewing the manuscript.

## Conflicts of interest

There are no conflicts to declare.

## Supplementary Material

EA-005-D5EA00041F-s001

## Data Availability

Gridded 2D data generated for this article available for download from the UCL Data Repository include top-down monthly total biomass burning NO_*x*_ and NH_3_ emissions (Fig. 4), monthly mean GEOS-Chem tropospheric columns of NO_2_ co-sampled with TROPOMI (Fig. 2), total columns of NH_3_ co-sampled with IASI (Fig. 3), and monthly mean IASI NH_3_ columns reprocessed with GEOS-Chem *a priori* profiles (Fig. 3) [https://doi.org/10.5522/04/28712444].
