# Supplementary material for: Subtropical southern Africa fire emissions of nitrogen oxides and ammonia obtained with satellite observations and GEOS-Chem
Source: Environ Sci Atmos. 2025 Jun 16;5(8):906–20. doi: 10.1039/d5ea00041f (PMC12180295; doi:10.1039/d5ea00041f)
Supplement: EA-005-D5EA00041F-s001 [file EA-005-D5EA00041F-s001.pdf]

Supplementary Information (SI) for Environmental Science: Atmospheres.
This journal is © The Royal Society of Chemistry 2025

Supporting Information (SI) for

Subtropical southern Africa fire emissions of nitrogen oxides and ammonia obtained with satellite observations and GEOS-Chem

Eloise A. Marais[a,*], Martin Van Damme[b,c], Lieven Clarisse[b], Christine Wiedinmyer[d], Killian Murphy[e], Guido R. van der Werf[f]

[a]Department of Geography, University College London, London, UK.

[b]Spectroscopy, Quantum Chemistry and Atmospheric Remote Sensing (SQUARES), BLU-ULB Research Center, Université libre de Bruxelles (ULB), Brussels, Belgium

[c]Royal Belgian Institute for Space Aeronomy (BIRA-IASB), Brussels, Belgium

[d]Cooperative Institute for Research in Environmental Sciences, University of Colorado, Boulder, Boulder, CO, USA.

[e]Wolfson Atmospheric Chemistry Laboratories, Department of Chemistry, University of York, York, UK.

[f]Meteorology & Air Quality Group, Wageningen University and Research, Wageningen, The Netherlands

*Corresponding author: Eloise A. Marais (e.marais@ucl.ac.uk)

Contents of this SI file:

Text S1-S2
Figures S1-S4

Text S1: Application of averaging kernels to GEOS-Chem for comparison to TROPOMI NO_2

The steps for applying TROPOspheric Monitoring Instrument (TROPOMI) averaging kernels to model vertical profiles of nitrogen dioxide (NO_2) are detailed in the TROPOMI Product User Manual. [1] Tropospheric averaging kernels ($A^{troposphere}_{TROPOMI}$) are first calculated by scaling total column averaging kernels ($A^{total}_{TROPOMI}$) by the ratio of total column air mass factors (AMF) (AMF^{total}) to the tropospheric column AMF ($AMF^{troposphere}$):

$$A^{troposphere}_{TROPOMI} = A^{total}_{TROPOMI} \frac{AMF^{total}}{AMF^{troposphere}} \qquad (S1)$$

The TROPOMI averaging kernels from Equation (S1) are then interpolated to the GEOS-Chem vertical grid and multiplied by model shape factors (the relative contribution of NO_2 in each GEOS-Chem vertical layer l) and summed over all troposphere layers to yield GEOS-Chem NO_2 vertical column densities (VCD) with TROPOMI vertical sensitivities:

$$VCD^{troposphere}_{GEOS-Chem} = \sum_l A^{troposphere}_{l,TROPOMI} x_{l,GEOS-Chem} \qquad (S2)$$

Text S2: IASI NH_3 reprocessing with local GEOS-Chem a priori profiles

IASI ammonia (NH_3) averaging kernels (A^{total}_{IASI}) are calculated by dividing the provided integrated IASI NH_3 total columns ($VCD^{total}_{IASI\ default}$) by the provided confined vertically resolved columns ($VCD^{confined}_{IASI}$):

$$A^{total}_{IASI} = \frac{VCD^{total}_{IASI\ default}}{VCD^{confined}_{IASI}} \qquad (S3)$$

The averaging kernels can also be normalized by a provided normalization factor [2], but we find that this only leads to discernible differences in monthly means for gridboxes with fewer than 10 IASI pixels. Reprocessed IASI NH_3 total VCDs ($VCD^{total}_{IASI\ reprocessed}$) are then calculated by dividing the reported $VCD^{total}_{IASI\ default}$ by the integrated product of the GEOS-Chem NH_3 shape factor and A^{total}_{IASI} interpolated to the GEOS-Chem vertical grid:

$$VCD^{total}_{IASI\ reprocessed} = \frac{VCD^{total}_{IASI\ default}}{\sum_l A^{total}_{l,IASI} x_{l,GEOS-Chem}} \qquad (S4)$$

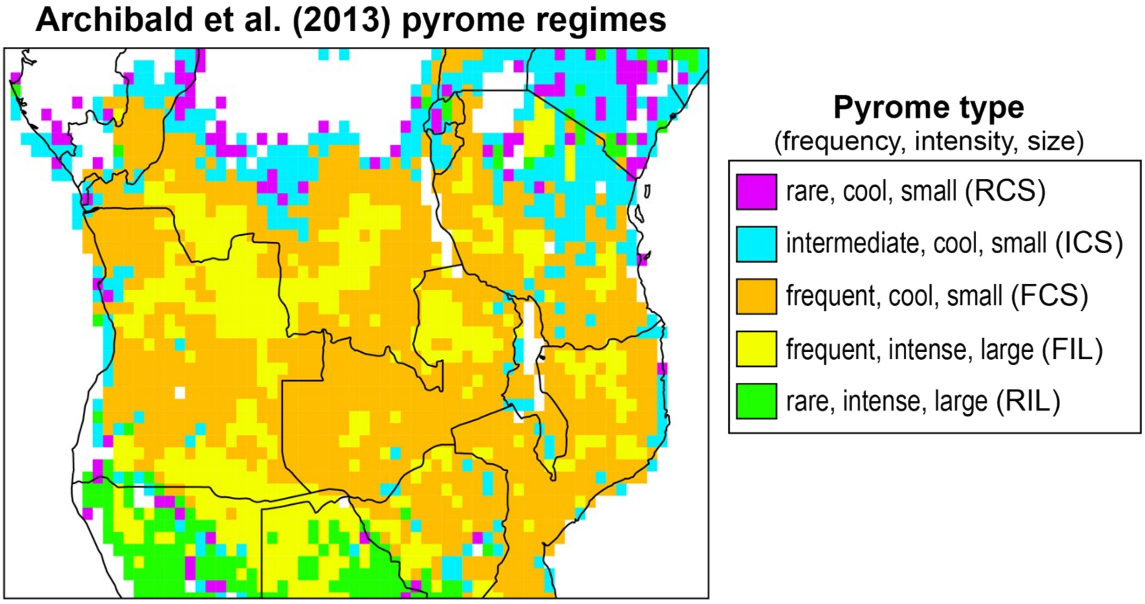

Figure S1. Archibald et al. (2013)[3] pyrome classes used to interpret the spatial distribution of top-down estimated nitrogen oxides (NO_x) and NH_3 emissions. Data are on a 0.5° grid. A brief description of the derivation of the dataset is in the main manuscript (Section 2.5). Data downloaded from the archive hosted by the Archibald Ecology Laboratory (https://archibaldlab.weebly.com/datasets.html, last accessed 25 February 2025) are visualised with the same colour scheme as Archibald et al. (2013)[3].

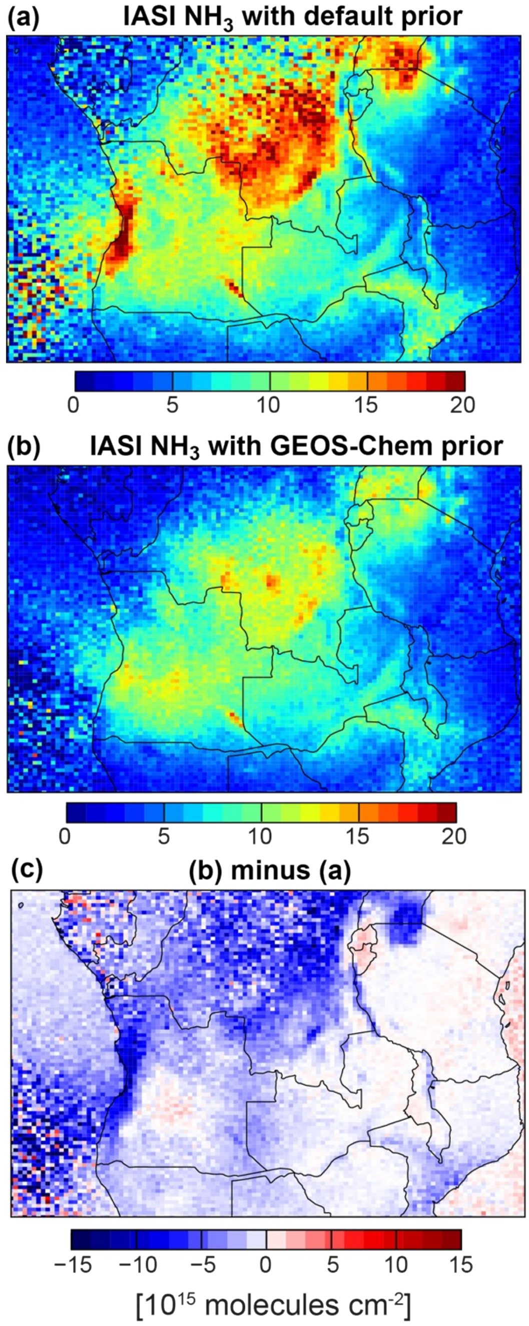

Figure S2. Effect of the GEOS-Chem prior on IASI NH_3. Panels are IASI NH_3 retrieved with a fixed land and ocean a priori profile (a), with local a priori profiles from GEOS-Chem (b), and the difference between the two (c). Data are July-October 2019 means on the GEOS-Chem 0.25° latitude × 0.3125° longitude grid.

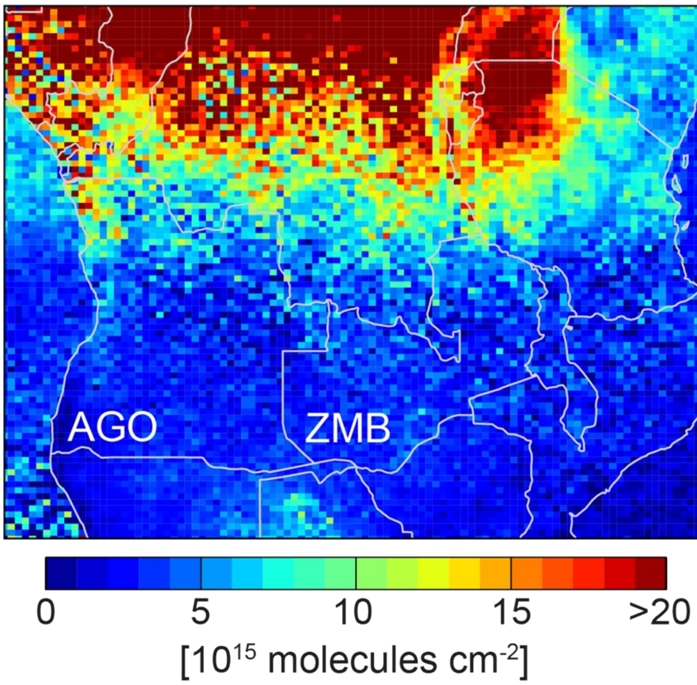

Figure S3. IASI NH_3 for non-biomass-burning months in southern Africa in 2019. Data shown are the provided December, January and February IASI NH_3 total columns obtained with the default static a priori. The widespread hotspot of IASI NH_3 ($>35 \times 10^{15}$ molecules cm^{-2}) in the northern portion of the domain is from biomass burning during the peak of the northern Africa burning season. The enhancements in NH_3 in southern Angola and on the Angola side of the border with Zambia in Figure 3(a) (Section 3.3) are both absent, supporting interpretation that these are from dry season fires. Angola (AGO) and Zambia (ZMB) are indicated.

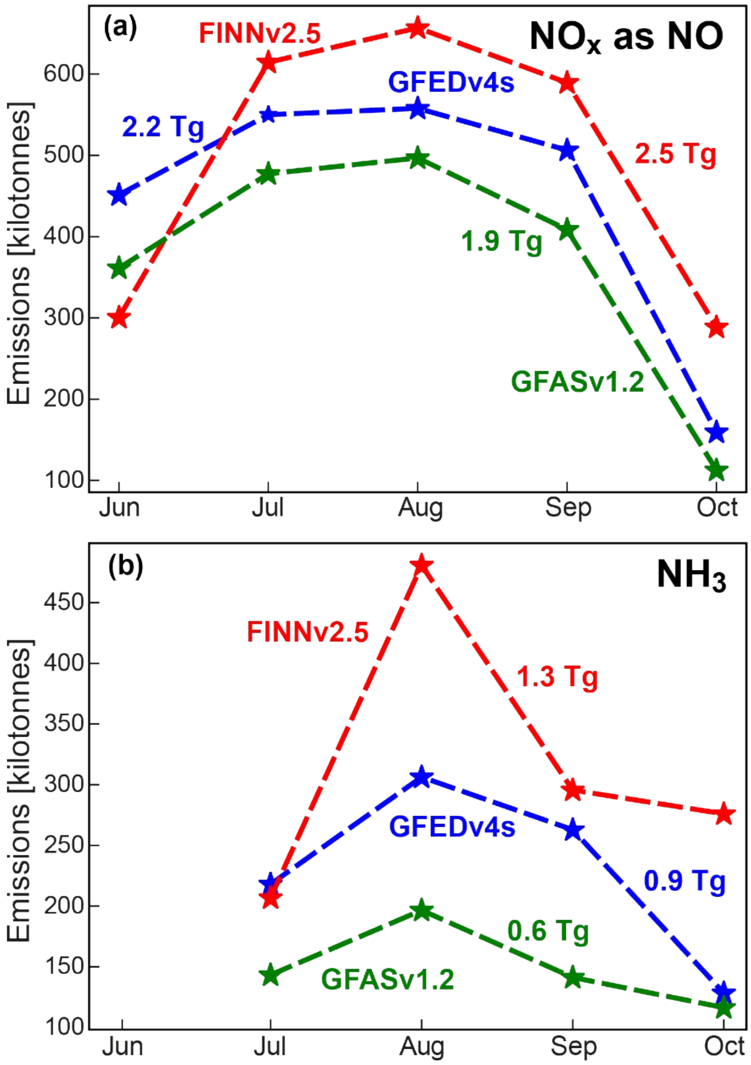

Figure S4. Sensitivity of monthly total top-down emissions to prior biomass burning inventories. Panels are top-down June-October NO_x emissions obtained using TROPOMI tropospheric columns of NO_2 (a) and July-October top-down NH_3 emissions obtained using IASI NH_3 (b). Colours discern top-down emissions estimates using GEOS-Chem driven with GFEDv4s (blue), FINNv2.5 (red), or GFASv1.2 (green). The manuscript details the mass balance emission inversion approach (Equation (3), Section 2.5) and the reason (b) excludes June.

References:

1. H. Eskes, J. v. Geffen, F. Boersma, K.-U. Eichmann, Arnoud Apituley, M. Pedergnana, M. Sneep, J. P. Veefkind and D. Loyola, Sentinel-5 precursor/TROPOMI Level 2 Product User Manual Nitrogendioxide, 2022.
2. L. Clarisse, B. Franco, M. Van Damme, T. Di Gioacchino, J. Hadji-Lazaro, S. Whitburn, L. Noppen, D. Hurtmans, C. Clerbaux and P. Coheur, The IASI NH_3 version 4 product: averaging kernels and improved consistency, *Atmos. Meas. Techn.*, 2023, **16**, 5009-5028.
3. S. Archibald, C. E. R. Lehmann, J. L. Gómez-Dans and R. A. Bradstock, Defining pyromes and global syndromes of fire regimes, *PNAS*, 2013, **110**, 6442-6447.
